# A Novel Approach to the Viability Determination of *Mycobacterium avium* subsp. *paratuberculosis* Using Platinum Compounds in Combination With Quantitative PCR

**DOI:** 10.3389/fmicb.2021.748337

**Published:** 2021-11-24

**Authors:** Martina Cechova, Monika Beinhauerova, Vladimir Babak, Iva Slana, Petr Kralik

**Affiliations:** ^1^Department of Microbiology and Antimicrobial Resistance, Veterinary Research Institute, Brno, Czechia; ^2^Department of Experimental Biology, Faculty of Science, Masaryk University, Brno, Czechia; ^3^Laboratory of Neurobiology and Pathological Physiology, Institute of Animal Physiology and Genetics, Czech Academy of Sciences, Libechov, Czechia

**Keywords:** viability, qPCR, live-dead discrimination, platinum, mycobacteria, *Mycobacterium avium* subsp. *paratuberculosis*, propidium monoazide

## Abstract

Mycobacterium avium subsp. *paratuberculosis* (MAP) represents a slow-growing bacterium causing paratuberculosis, especially in domestic and wild ruminants. Until recently, the assessment of MAP viability relied mainly on cultivation, which is very time consuming and is unable to detect viable but non-culturable cells. Subsequently, viability PCR, a method combining sample treatment with the DNA-modifying agent ethidium monoazide (EMA) or propidium monoazide (PMA) and quantitative PCR (qPCR), was developed, enabling the selective detection of MAP cells with an intact cell membrane. However, this technology requires a laborious procedure involving the need to work in the dark and on ice. In our study, a method based on a combination of platinum compound treatment and qPCR, which does not require such a demanding procedure, was investigated to determine mycobacterial cell viability. The conditions of platinum compound treatment were optimized for the fast-growing mycobacterium *M. smegmatis* using live and heat-killed cells. The optimal conditions consisting of a single treatment with 100 μM *cis*-dichlorodiammine platinum(II) for 60 min at 5°C resulted in a difference in quantification cycle (Cq) values between live and dead membrane-compromised mycobacterial cells of about 6 Cq corresponding to about 2 log_10_ units. This optimized viability assay was eventually applied to MAP cells and demonstrated a better ability to distinguish between live and heat-killed mycobacteria as compared to PMA. The viability assay combining the Pt treatment with qPCR thereby proved to be a promising method for the enumeration of viable MAP cells in foodstuffs, environmental, and clinical samples which could replace the time-consuming cultivation or laborious procedures required when using PMA.

## Introduction

The detection of mycobacteria in recent years relied mainly on cultivation-based methods. However, these are only able to detect viable mycobacteria if they are cultivable, which may represent a substantial deficiency of these conventional methods. The number of mycobacteria detected by the culture may be reduced by those present in a dormant or viable non-culturable state (Elguezabal et al., 2011). In addition, the cultivation of slow-growing mycobacterial species such as *Mycobacterium avium* subsp. *paratuberculosis* (MAP), which is the causative agent of paratuberculosis, particularly in domestic and wild ruminants is very time consuming (several weeks to months). Another disadvantage of MAP cultivation is the need for chemical decontamination of the sample to suppress the growth of competitive microorganisms, which can result in a decrease in the sensitivity of detection (Slana et al., 2008). Moreover, the number of MAP cells may be underestimated due to the tendency of MAP cells to form clumps (Pickup et al., 2005).

The introduction of quantitative PCR (qPCR) has overcome these limitations, as qPCR is fast, more sensitive, and capable of detecting and quantifying even non-cultivable bacteria. qPCR systems targeting the multicopy element IS*900* and the single copy element *F57* have been developed for the specific detection and quantification of MAP, respectively (Slana et al., 2008). However, the disadvantage of qPCR is the inability to distinguish live bacteria from dead ones (live-dead discrimination), which makes this method unsuitable for assessing MAP viability. In past years, DNA intercalating dyes—ethidium monoazide (EMA) and propidium monoazide (PMA)—have been shown to enable viability determination in combination with PCR (Nogva et al., 2003; Nocker et al., 2006). These dyes penetrate membrane-compromised non-viable cells in which they form a covalent link to DNA, thereby suppressing the target nucleic acid amplification in qPCR following the cell treatment and genomic DNA extraction. Conversely, EMA and PMA do not permeate into live bacteria with an intact cell membrane, or do so to only a limited extent. An approach combining qPCR with the intercalation dyes EMA or PMA has already been applied to a wide range of gram-negative and gram-positive bacteria (Nocker and Camper, 2009). Furthermore, PMA-qPCR has also been used for the evaluation of the killing efficiency by disinfection or heat, both of which cause cell membrane damage (Nocker et al., 2007). PMA-qPCR has also been introduced for viable MAP cell quantification targeting *F57* (Kralik et al., 2010). The disadvantage of EMA/PMA-qPCR is the laborious procedure involving the need to work in the dark, since these compounds are activated by visible light. It is also necessary to keep the bacterial suspension on ice to avoid disruption of the cell wall or membrane of live cells due to an increase in temperature from light exposure with a halogen lamp, which may subsequently result in the undesirable penetration of the agents into live cells (Fittipaldi et al., 2012). This has led to the search for other compounds that enable live-dead discrimination without the need for such a demanding procedure.

Lately, platinum (Pt) and palladium (Pd) compounds—e.g., dichloro(ethylenediamine) platinum(II), *cis*-dichlorodiammineplatinum (II), platinum (IV) chloride, palladium(II) acetate, bis(benzonitrile) dichloropalladium(II), trans-diammine dichloropalladium(II)—have proven to be suitable candidates for viability assays. Their great advantage is that they are not as highly sensitive to visible light as EMA and PMA, meaning that working with them does not require a darkroom, while it is also not necessary to work on ice, and their effect is not conditioned by excitation by light. In addition, the Pt and Pd agents are less expensive than the monoazide dyes (Soejima and Iwatsuki, 2016; Soejima et al., 2016). These agents, like the aforementioned intercalating dyes, generally penetrate dead cells with a compromised membrane but not live ones, for which reason viability detection is also based on membrane integrity. Pt and Pd complexes within cells interact with DNA resulting in either intrastrand (between the bases on the same chain) or interstrand (between bases on opposite chains) cross-links, which subsequently interfere with the binding of DNA polymerase to the DNA and the amplification of the target nucleic acid sequence. Therefore, when detecting bacterial viability, only unmodified DNA derived from live cells is amplified during qPCR (Soejima et al., 2016). Pt and Pd compounds in conjunction with qPCR have already been applied in the detection and quantification of viable bacterial cells, *Escherichia coli* and *Cronobacter sakazakii* (Soejima and Iwatsuki, 2016; Soejima et al., 2016). Subsequently, these agents combined with reverse transcription qPCR (RT-qPCR) were also used for evaluation of viral infectivity, which is in turn based on capsid integrity, such as with human norovirus (NoV) and murine norovirus (MNV) (Fraisse et al., 2018), hepatitis E virus (HEV) and hepatitis A virus (HAV) (Randazzo et al., 2018), porcine epidemic diarrhea virus (PEDV) (Puente et al., 2020), and Aichi virus (Canh et al., 2019).

In this study, we evaluated the use of Pt compounds combined with qPCR for the discrimination of live and heat-killed (membrane-compromised) mycobacterial cells. To this end, five Pt compounds were screened, and treatment conditions were optimized for the fast-growing mycobacterium *M. smegmatis*. The optimized viability assay conditions using Pt compounds were finally applied to MAP and compared with PMA treatment as the reference method for MAP viability determination.

## Materials and Methods

### Bacterial Strains and Culture Conditions

Optimization of treatment conditions with Pt compounds was performed with the fast-growing *M. smegmatis* strain (collection strain ATCC 700084). The optimized conditions were applied to slow-growing MAP (field isolate 7072). The strains were grown on Herrold’s egg yolk medium (HEYM), for MAP in addition with 2 μg/ml of Mycobactin J (Allied Monitor, United States) (HEYM-MJ), and incubated at 37°C. Both mycobacterial cultures grown were then inoculated into liquid Middlebrook 7H9 broth (Difco, Livonia, United States), supplemented with Middlebrook OADC enrichment (Difco), and in the case of MAP also with the addition of 2 μg/ml of Mycobactin J, and cultured at 37°C with shaking to attain an optical density at 600 nm (OD_600_) of about 1.0 (BioPhotometer; Eppendorf, Germany). The bacterial cultures were centrifuged at 3,000 × g for 5 min, and the supernatant was discarded and replaced with ultrapure water (Top-Bio, Czech Republic). The bacterial suspensions were homogenized using vortex and 1 mm zirconia beads (BioSpec, United States) and centrifuged at 100 × g for 30 s to remove big clumps. The upper fraction of the cell suspension was diluted with ultrapure water to an OD_600_ of about 0.15, which corresponded to 10^7^ cells/ml, as determined by subsequent qPCR. The bacterial suspension was distributed in an amount of 500 μl per microtube. One half of the 500 μl aliquots of bacterial suspension was heat-treated (see below) and represented “dead cells,” while the other non-heat-treated half of the aliquots constituted “live cells.”

The amount of viable *M. smegmatis* cells in the bacterial suspension was assessed by culturing, spreading 100 μl of an appropriate dilution of the suspension in triplicates on plates with the HEYM medium, and incubating at 37°C for 3 days until colony forming unit (CFU) counting.

### Preparation of Dead Cells by Heat Treatment

Heat treatment was conducted for both *M. smegmatis* and MAP by exposing the 500 μl aliquots of bacterial suspensions to 100°C for 4 min with shaking (100 rpm) using heat block and immediately cooling on ice. Killing efficacy was verified by seeding the heat-treated *M. smegmatis* and MAP cells on HEYM and HEYM-MJ agar, respectively, and incubating at 37°C for 2 and 4 weeks, respectively. Based on no difference recorded in the Cq values of heat-killed and non-heat-treated (both Pt-untreated) *M. smegmatis* and MAP cells in qPCR, it was shown that the heat treatment did not cause cell rupture and, thereby, DNA release and loss. This dead cell preparation protocol was part of each of the experiments performed in section “Viability Assay on Mycobacterial Cells.”

### Preparation of Platinum Compounds and Propidium Monoazide

Five Pt compounds, previously described by Soejima et al. (2016), were used for viability testing: dichloro(ethylenediamine) platinum (II), *cis*-dichlorodiammineplatinum (II), tetrakis(triphenylphosphine) platinum (0), chloroplatinic acid hexahydrate, and platinum (IV) chloride (Sigma-Aldrich, United States). The chemicals were dissolved and diluted to appropriate concentrations (specified for individual experiments below) in physiological saline and left for around 1 h at 40°C with shaking (1,000 rpm) in the dark to promote dissolution of the compounds. Pt compounds were not dissolved in the organic solvent dimethyl sulfoxide (DMSO) as it is discouraged due to their possible interaction altering the structure of the Pt compound complexes and impairing their ability to interact with DNA (Yi and Bae, 2011; Hall et al., 2014).

PMA was prepared based on the previous study by Kralik et al. (2010), in which 1 mg of PMA (Biotium, United States) was dissolved in 1.9 ml of 20% DMSO (Sigma-Aldrich) to obtain a 1 mM stock solution.

### Direct Effect of Platinum Compounds on *Mycobacterium avium* subsp. *paratuberculosis* DNA Amplification

**FIGURE 1:**
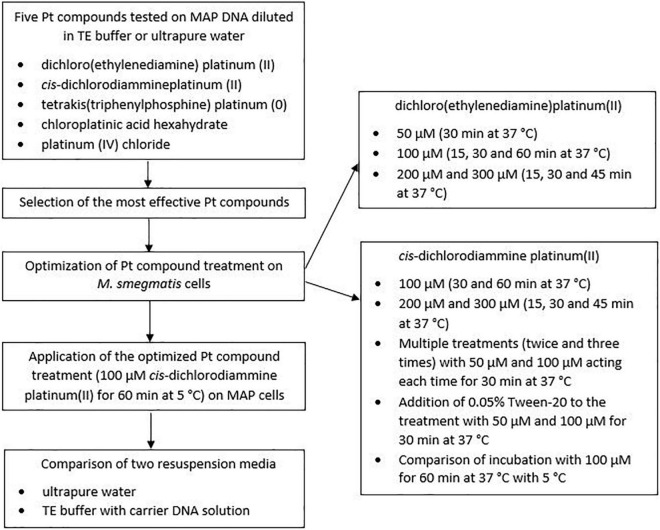
Schematic overview of the optimization of Pt compound treatment.

One loop of grown MAP culture was resuspended in 200 μl of ultrapure water. MAP DNA was then isolated using a Quick-DNA Fecal/Soil Microbe Microprep kit (Zymo Research, Tustin, California, United States) according to manufacturer’s protocol. A MagNA Lyser instrument (Roche Diagnostics GmbH, Mannheim, Germany) was used for disruption of the MAP cells, allowing the tubes to be shaken at 6,400 rpm for 1 min. The concentration of isolated DNA was determined using a NanoDrop 2000c spectrophotometer (Thermo Fisher Scientific, United States). DNA purity was checked to assure that the ratio of the absorbance at 260 and 280 nm (A_260/280_) was not higher than 1.8 in order to avoid significant contamination with RNA. The DNA was divided into two portions. The first was diluted with ultrapure water and the second with a Tris-ethylenediaminetetraacetic acid (EDTA; TE) buffer (Serva Electrophoresis GmbH, Germany) to a concentration of 1 ng/μl and distributed in an amount of 50 μl per 2 ml microtube. Ten microliter of each Pt compound solution diluted to the appropriate concentration (10, 100, and 1,000 μM in the final DNA solution) was then added individually to the microtube lids, which were subsequently gently closed, and the Pt compounds and DNA solutions were uniformly mixed by a short spin. After incubation with a particular agent for 30 min at 37°C with shaking (100 rpm), MAP DNA was purified using a DNeasy Blood & Tissue Kit (Qiagen, Germany), after which qPCR was performed. In the case of control samples, the same procedure was followed except for the addition of 10 μl of physiological saline instead of the Pt compound solution. The experiment was carried out in biological duplicates for each condition. A schematic overview of the optimization of Pt compound treatment is shown in Figure 1.

### Viability Assay on Mycobacterial Cells

#### Optimization of the Viability Assay Using Platinum Compounds on *M. smegmatis*

Ten μl of Pt compound solutions diluted to appropriate concentrations corresponding to 50, 100, 200, and 300 μM in the final bacterial suspension was added individually to the lids of the microtubes with bacterial suspensions of live and dead cells (500 μl) prepared as described in section “Preparation of Dead Cells by Heat Treatment.” For controls, an identical volume of physiological saline was added instead of the Pt compound solution. The microtube lids were gently closed, and the Pt-treated and non-treated bacterial suspensions were uniformly spun briefly and incubated for an appropriate time (15, 30, 45, or 60 min depending on the concentration used) at 37°C with shaking (100 rpm). All the microtubes were then centrifuged at 7,000 × g for 5 min. The supernatant was discarded and the cell pellet was subjected to DNA extraction (see below). Each tested condition was analyzed in biological duplicate.

In order to increase the difference in quantification cycle (Cq) values between live and dead cells, multiple treatments (twice and three times with intermediate harvesting of cells by centrifugation) with Pt compound solutions [50 and 100 μM *cis*-dichlorodiammine platinum(II) in the final bacterial suspension] acting each time for 30 min were also applied. Another factor investigated to increase the difference in Cq values between live and dead cells was the use of a surfactant (0.05% Tween-20; VWR Chemicals, United States) that was added to 500 μl aliquots of viable and dead cell suspensions immediately prior to the treatment with Pt compound solutions [50 and 100 μM *cis*-dichlorodiammine platinum(II) in the final bacterial suspension]. In both cases, all other steps were identical to the above procedure.

The last optimization experiment concerned testing the effect of incubation temperature during Pt treatment, comparing 37°C (with shaking at 100 rpm) with 5°C (manually shaken with all microtubes every 15 min during the incubation).

#### Application of the Optimized Viability Assay Using Platinum Compound on *Mycobacterium avium* subsp. *paratuberculosis*

The conditions optimized in the viability assay on *M. smegmatis* were subsequently applied to MAP. The optimized procedure consisted of a single treatment of live and dead MAP cells with 100 μM *cis*-dichlorodiammine platinum(II) for 60 min at 5°C (with occasional manual shaking) without the use of the surfactant followed by centrifugation at 7,000 × g for 5 min, removal of supernatant, and DNA extraction.

#### Viability Assay Using Propidium Monoazide

The procedure of PMA treatment was performed according to the study by Kralik et al. (2010). Briefly, under minimal light conditions, 12.5 μl of PMA was added to 500 μl aliquots of viable and dead MAP cell suspensions to attain a final concentration of 25 μM, the microtubes were briefly centrifuged and incubated at room temperature for 5 min with shaking (1,200 rpm). The microtubes were then placed horizontally on ice and exposed to light from a halogen lamp with a 650 W bulb from a distance of about 20 cm for 2 min. The PMA treatment step was repeated once more. Subsequently, the microtubes were centrifuged at 7,000 × g for 5 min, the supernatant was discarded, and the cell pellets subjected to DNA extraction. The same procedure was carried out for control samples, but instead of PMA the same volume of 20% DMSO was added to 500 μl aliquots of viable and dead MAP cell suspensions. Each tested condition was analyzed in biological duplicate.

### DNA Extraction Following the Platinum and Propidium Monoazide Treatment on Mycobacterial Cells

Mycobacterial DNA was obtained as a crude lysate according to the study by Kralik et al. (2010) which reported that the crude lysate preparation proved to be a suitable alternative to commercial DNA isolation kits. For *M. smegmatis*, pellets of Pt-treated and untreated cells (live and dead cells in both cases) were resuspended in 500 μl of ultrapure water. In the case of MAP, the effect of ultrapure water and TE buffer supplemented with carrier DNA solution (salmon sperm DNA, 50 ng/μl; Serva, Germany) that reduces DNA losses during manipulation (Slana et al., 2008) in a volume of 500 μl used to resuspend the pellets of Pt- or PMA-treated and untreated cells was monitored. The resuspended cells were then subjected to lysis at 100°C for 20 min. Subsequently, centrifugation at 14,000 × g for 5 min was performed and the supernatant containing the DNA was analyzed by qPCR.

### Quantitative PCR and Data Analysis

DNA purification (in the case of the application of Pt compounds directly to MAP DNA) or extraction (in the viability assay on mycobacterial cells) were followed by qPCR. For *M. smegmatis*, a qPCR assay targeting the ITS (internal transcribed spacer) sequence was performed as previously described by Sevilla et al. (2015). qPCR targeting the *F57* sequence developed by Slana et al. (2008) was used for MAP. Both qPCR assays included an internal amplification control (IAC) that allowed false negative results to be detected. All qPCR reactions were performed using a LightCycler 480 (Roche Molecular Diagnostic, Germany). All qPCR samples were tested in technical duplicates.

Cq values were determined using the “Fit Point Analysis” option of the LightCycler 480 software (version 1.5.0.39). The differences between the Cq values (ΔCq) of isolated MAP DNA or live and dead cells treated with Pt compounds or PMA and the respective untreated control samples calculated from all biological and technical replicates were compared using the following formulas, which were taken from Kralik et al. (2010) and slightly modified:

(1) $$\Delta \,{\text{Cq}}_{\mathrm{DNA}\,\mathrm{with}\,\mathrm{Pt}-\mathrm{DNA}\,\mathrm{without}\,\mathrm{Pt}}$$

(2) $$\Delta \,{\text{Cq}}_{\mathrm{dead}\,\mathrm{with}\,\mathrm{Pt}/\mathrm{PMA}-\mathrm{live}\,\mathrm{with}\,\mathrm{Pt}/\mathrm{PMA}}$$

(3) $$\Delta \,{\text{Cq}}_{\mathrm{dead}\,\mathrm{with}\,\mathrm{Pt}/\mathrm{PMA}-\mathrm{dead}\,\mathrm{without}\,\mathrm{Pt}/\mathrm{PMA}}$$

(4) $$\Delta \,{\text{Cq}}_{\mathrm{live}\,\mathrm{with}\,\mathrm{Pt}/\mathrm{PMA}-\mathrm{live}\,\mathrm{without}\,\mathrm{Pt}/\mathrm{PMA}}$$

Equation (1) expresses the differences in target sequence amplification of Pt-treated and untreated DNA samples. The highest possible value is desirable, indicating that the Pt compound binds significantly to the target DNA sequence and thus prevents its amplification during qPCR. Equation (2) expresses the extent to which a particular Pt or PMA permeates dead cells while taking into account the undesired permeation of the agents into live cells. Equation (3) also expresses the extent to which Pt or PMA permeates dead cells, regardless of live cells. For both Equations (2) and (3), the highest possible value is desirable (ideally no amplification in dead Pt- or PMA-treated cells indicating that the agents bound to all target sequences and thus prevented their amplification in all dead cells). Equation (4) expresses the extent to which Pt or PMA permeates live cells, so the lowest possible value is required.

### Statistical Analysis

Analysis of ΔCq was performed by one-way analysis of variance (ANOVA), two-way ANOVA, Welch’s *t*-test, and Tukey’s HSD test (detailed in the relevant figures). Data analysis was performed using the statistical software Statistica 13.2 (StatSoft Inc., Tulsa, OK, United States). *P*-values less than 0.05 were considered statistically significant.

## Results

### Direct Effect of Platinum Compounds on *Mycobacterium avium* subsp. *paratuberculosis* DNA Amplification

**FIGURE 2:**
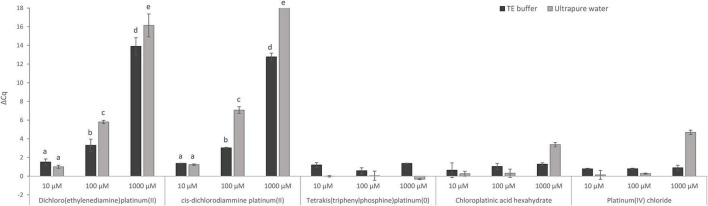
Direct effect of Pt compound solutions at various concentrations on amplification of MAP DNA diluted with TE buffer or ultrapure water. The ΔCq values express the mean difference of individual Cq values of Pt-treated and mean Cq value of untreated control samples calculated from four replicates (biological and technical). The significance of differences between ΔCq was calculated by two-way ANOVA with factors of concentration and dilution medium and Tukey’s HSD test. The same letters indicate statistically insignificant differences (*P* > 0.05) between ΔCq values, while different letters indicate significant differences (*P* < 0.05). Error bars represent standard deviations calculated from four replicates. The bar above value of 18 in ΔCq on the *y*-axis signifies that no amplification occurred.

First, the direct effect of five Pt compounds on the amplification of a target sequence of isolated MAP DNA was evaluated, wherein all Pt compounds were tested at three concentrations: 10, 100, and 1,000 μM in DNA solution (Figure 2). The aim of this experiment was to select a Pt compound that shows maximal binding to the target DNA sequences and thus prevents its amplification during qPCR without affecting the IAC. The differences in the Cq values of Pt-treated and untreated samples were expressed according to Equation (1). Significant suppression of MAP DNA amplification was observed for two Pt compounds: dichloro(ethylenediamine)platinum(II) and *cis*-dichlorodiammine platinum(II). Reduction of the qPCR signals was, as expected, concentration-dependent. At a concentration of 1,000 μM, a significant signal reduction of about 16 Cq and complete amplification suppression was achieved for dichloro(ethylenediamine)platinum(II) and *cis*-dichlorodiammine platinum(II), respectively, while a concentration of 100 μM already showed a lower reduction of about 6 and 7 Cq, respectively. The concentration of 10 μM was already insufficient for the Pt compound molecules to be chelated to a significant number of DNA template molecules. The other three Pt compounds [tetrakis(triphenylphosphine)platinum(0), chloroplatinic acid hexahydrate and platinum(IV) chloride] showed a limited effect on MAP DNA amplification (ΔCq < 5) even at the highest concentration tested (1,000 μM), for which reason the treatment with these compounds was excluded from the following optimization on mycobacterial cells.

In this experiment, the effect of TE buffer or ultrapure water on the activity of Pt compounds was also assessed (Figure 2). When TE buffer was used, a significantly lower reduction in amplification (lower ΔCq _DNA with Pt–DNA without Pt_) of the target DNA sequence after treatment with 100 and 1,000 μM dichloro(ethylenediamine)platinum(II) and *cis*-dichlorodiammine platinum(II) was observed compared to ultrapure water. Ultrapure water was, therefore, used in the following experiments as a dilution medium when diluting the mycobacterial cells.

After treatment of MAP DNA with Pt compounds, it was necessary to remove unbound Pt molecules from the DNA solution using a purification kit (DNeasy Blood & Tissue Kit) before performing qPCR. This necessity was demonstrated by the fact that the omission of the purification step considerably inhibited the amplification of IAC (data not shown).

### Optimization of the Viability Assay Using Platinum Compounds on *M. smegmatis*

**FIGURE 3:**
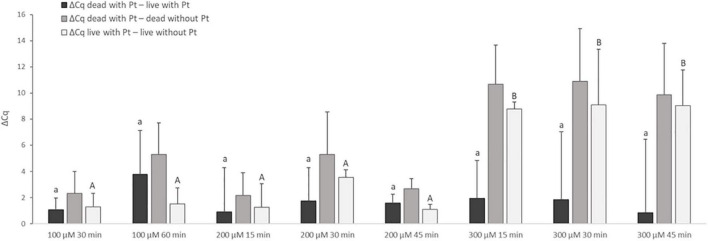
Effect of treatment of *M. smegmatis* cells with *cis*-dichlorodiammine platinum(II) at different concentrations and for different times. The aim was to find the treatment condition providing the highest possible value for ΔCq _dead with Pt_ _– live with Pt_ and ΔCq _dead with Pt_ _– dead without Pt_ and the lowest possible value for ΔCq _live with Pt_ _– live without Pt_. The ΔCq values express the individual mean differences of Cq values calculated from four replicates (biological and technical). The significance of differences between ΔCq was calculated by one-way ANOVA and Tukey’s HSD test. The same letters indicate statistically insignificant differences (*P* > 0.05) between ΔCq values, while different letters indicate significant differences (*P* < 0.05). The values of ΔCq _dead with Pt – live with Pt_ were marked with lowercase letters, and the values of ΔCq _live with Pt – live without Pt_ were marked with capital letters, as it was evaluated separately. Error bars represent standard deviations calculated from four replicates.

Treatment with Pt compounds that exhibited the greatest effect on MAP DNA amplification was subsequently optimized on live and dead *M. smegmatis* cells. The cells were treated with dichloro(ethylenediamine)platinum(II) and *cis*-dichlorodiammine platinum(II) at three concentrations for different exposure times (100 μM for 30 and 60 min; 200 and 300 μM for 15, 30, and 45 min) at 37°C. The differences in Cq values of live and dead cells treated with Pt compounds and the respective untreated control samples were calculated according to Equation (2–4). Dichloro(ethylenediamine)platinum(II) penetrated considerably into live cells at all three concentrations tested, as shown by the calculation of ΔCq _live with Pt – live without Pt_, whose lowest calculated value was about 7 Cq (data not shown). For *cis*-dichlorodiammine platinum(II), although not with a significant difference, the highest mean value of ΔCq _dead with Pt_ _– live with Pt_ was recorded at 100 μM, to which the cells were exposed for 60 min (Figure 3). Treatment with both 200 and 300 μM *cis*-dichlorodiammine platinum(II) did not lead to a higher value of ΔCq _dead with Pt_ _– live with Pt_, and in addition the 300 μM concentration caused more significant penetration of the agent into live cells. Based on these data, the use of *cis*-dichlorodiammine platinum(II) at concentrations greater than 100 μM was not found to be beneficial.

Since even a 100 μM concentration of dichloro(ethylenediamine)platinum(II) for 30 min considerably affected live cells, a lower concentration of 50 μM for 30 min and 100 μM for only 15 min was subsequently applied. Neither led to any improvement, i.e., lower penetration of this Pt compound into live cells (data not shown).

In order to try to increase ΔCq between live and dead cells, multiple treatments (twice and three times) with 50 and 100 μM *cis*-dichlorodiammine platinum(II) acting each time for 30 min were evaluated. In addition, the possibility of using a surfactant was also examined by adding 0.05% Tween-20 to the procedure involving a single exposure to 50 and 100 μM *cis*-dichlorodiammine platinum(II) for 30 min. However, both alterations repeatedly (in two independent experiments) resulted in an undesirable noticeable increase of Cq values for live cells (increase in ΔCq _live with Pt–live without Pt_) (data not shown). Therefore, one exposure of the cells to 100 μM *cis*-dichlorodiammine platinum(II) for 60 min without the use of the surfactant was chosen for subsequent experiments.

**FIGURE 4:**
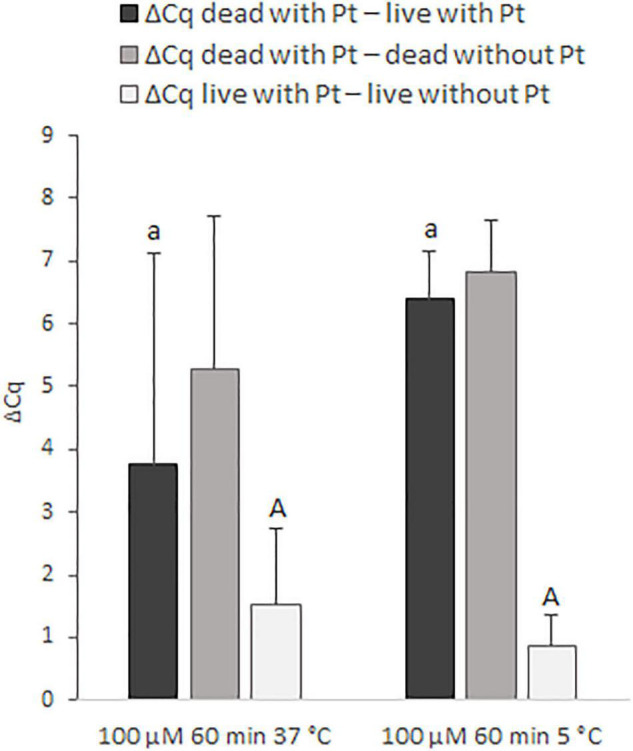
Effect of different incubation temperatures (37 and 5°C) on the treatment of *M. smegmatis* cells with 100 μM cis-dichlorodiammine platinum(II) for 60 min. The aim was to find the incubation temperature providing the highest possible value for ΔCq _dead with Pt__–__live with_ _Pt_ and ΔCq _dead with Pt__–__dead without Pt_ and the lowest possible value for ΔCq _live with Pt__–__live without Pt_. The ΔCq values express the individual mean differences of Cq values calculated from four replicates (biological and technical). The significance of differences between ΔCq was calculated by Welch’s *t*-test. The same letters indicate statistically insignificant differences (*P* > 0.05) between ΔCq values, while different letters indicate significant differences (*P* < 0.05). The values of ΔCq _dead with Pt – live with Pt_ were marked with lowercase letters, and the values of ΔCq _live with Pt – live without Pt_ were marked with capital letters, as it was evaluated separately. Error bars represent standard deviations calculated from four replicates.

In the next step, the effect of incubation temperature (37 and 5°C) on the discrimination of live and dead cells was assessed for 100 μM *cis*-dichlorodiammine platinum(II) (Figure 4). Incubation of the cells for 60 min with this Pt compound at 5°C provided higher mean values of ΔCq _dead_ _with_ _Pt__–__live_ _with_ _Pt_ (by about 2.7 Cq), as well as ΔCq _dead_ _with_ _Pt – dead without Pt_ (by about 1.6 Cq), and a lower mean value of ΔCq _live with Pt – live without Pt_ (by about 0.7 Cq) compared to incubation at 37°C. Although these differences in ΔCq values were not statistically significant, based on more favorable mean ΔCq values and standard deviations, it was evaluated that incubation at 5°C was superior to 37°C.

By evaluating all the conditions tested for *M. smegmatis*, it was concluded that a single treatment with 100 μM *cis*-dichlorodiammine platinum(II) for 60 min at 5°C without the use of the surfactant made it possible to attain the highest value of ΔCq _dead with Pt – live with Pt_ and at the same time the lowest value of ΔCq _live with Pt – live without Pt_. Finally, the optimized procedure for this Pt compound was applied to MAP.

### Application of the Optimized Viability Assay Using Platinum Compounds on *Mycobacterium avium* subsp. *paratuberculosis*

**FIGURE 5:**
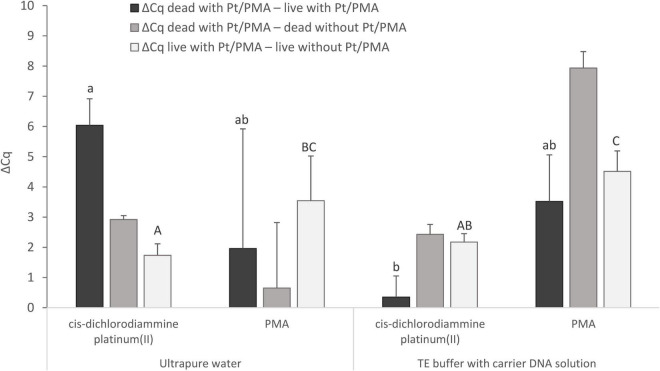
Comparison of Pt and PMA treatment of MAP cells and the effect of different resuspension media (ultrapure water or TE buffer with carrier DNA solution) used to resuspend the cells after the treatment. The aim was to determine the agent showing the highest possible value for ΔCq _dead with Pt/PMA__–__live with Pt/PMA_ and ΔCq _dead with Pt/PMA__–__dead without Pt/PMA_ and the lowest possible value for ΔCq _live with Pt/PMA__–__live without Pt/PMA_. The ΔCq values express the individual mean differences of Cq values calculated from four replicates (biological and technical). The significance of differences between ΔCq was calculated by two-way ANOVA with factors of resuspension medium and agent used, and Tukey’s HSD test. The same letters indicate statistically insignificant differences (*P* > 0.05) between ΔCq values, while different letters indicate significant differences (*P* < 0.05). The values of ΔCq _dead with Pt – live with Pt_ were marked with lowercase letters, and the values of ΔCq _live with Pt – live without Pt_ were marked with capital letters, as it was evaluated separately. Error bars represent standard deviations calculated from four replicates.

Live and dead MAP suspensions were subjected to the optimized Pt treatment condition as described above, which was compared with PMA treatment as the reference method for MAP viability determination (Figure 5). The differences in Cq values of live and dead cells treated with Pt or PMA compounds and the respective untreated control samples were calculated according to Equations (2–4).

For MAP, when *cis*-dichlorodiammine platinum(II) was applied and ultrapure water was used as the resuspension medium, ΔCq _dead with Pt – live with Pt_ gave a result of slightly above 6 Cq and ΔCq _live with Pt – live without Pt_ of about 1.5 Cq, which corresponded approximately to the values obtained with *M. smegmatis*. However, a markedly low ΔCq _dead with Pt – dead without Pt_ was noted, which stemmed from lower DNA amplification in a control sample of dead cells, which was not observed in any of the optimization experiments in *M. smegmatis*. The cause could not be the rupture of the cells during the heat treatment, as in the case of control samples of dead cells in which TE buffer with carrier DNA solution was used as the resuspension medium, the same initial steps including heat treatment were applied and the amplification did not decrease. On the other hand, the use of TE buffer with carrier DNA solution in the Pt treatment of cells led to a significant undesirable decrease in the value of ΔCq _dead with Pt – live with Pt_ by about 6 Cq to almost zero compared to the use of ultrapure water. This result showed that the use of TE buffer with carrier DNA solution as a resuspension medium is not suitable in the treatment of cells with Pt compounds.

Regarding the use of PMA and a comparison of both resuspension media mentioned, both ΔCq _dead with Pt – live with Pt_ and ΔCq _dead with Pt – dead without Pt_ showed higher mean values with lower standard deviations when TE buffer with carrier DNA solution was used. However, significant undesired penetration of PMA into viable MAP cells was recorded in both cases, as shown by ΔCq _live with Pt – live without Pt_ (about 4 Cq). Based on these data, it can be suggested that *cis*-dichlorodiammine platinum(II) appeared to be more suitable for distinguishing between viable and dead membrane-compromised MAP cells than PMA.

## Discussion

The present study aimed to optimize the viability determination of mycobacterial cells using Pt compounds in combination with qPCR. The procedure of Pt compound treatment is conditioned by disruption of cell membranes in dead cells (e.g., by high temperature) into which the agents can penetrate, while viable cells with intact membranes remain unaffected. Inside the membrane-compromised dead cells, the Pt compounds interact with DNA and subsequently interfere with the amplification of the target nucleic acid sequence during qPCR. qPCR, which follows the Pt-treatment, thereby enables the evaluation of the differences between Pt-treated and untreated control samples through measured Cq values. The viability assay combining the sample treatment with Pt compounds and qPCR is able to detect the viable mycobacterial cells within 1 day and is therefore clearly faster than cultivation, a gold standard for viability evaluation, which is very challenging for slow-growing bacteria such as MAP.

In our study, five Pt compounds were first assessed to determine chelating ability with purified MAP DNA (Figure 2). The greatest suppression of MAP DNA amplification was observed for the Pt compounds dichloro(ethylenediamine)platinum(II) and *cis*-dichlorodiammine platinum(II), so these two Pt compounds were selected for further experiments. The Pt compounds dichloro(ethylenediamine)platinum(II) and *cis*-dichlorodiammine platinum(II) have already been confirmed to prevent nucleic acid amplification by the study by Soejima et al. (2016), in which these agents were applied to extracted DNA from *C. sakazakii*. Another study also examined the effect of these Pt compounds on purified RNA from NoV and MNV, and revealed that *cis*-dichlorodiammine platinum(II), platinum(IV) chloride, and tetrakis(triphenylphosphine)platinum(0) reduced nucleic acid amplification, while dichloro(ethylenediamine)platinum(II) had a very limited effect (Fraisse et al., 2018). Further studies have reported that platinum(IV) chloride can also bind to purified RNA from the HEV virus (Randazzo et al., 2018), and both platinum(IV) chloride and *cis*-dichlorodiammine platinum(II) can be chelated to purified RNA from PEDV (Puente et al., 2020).

Further, we investigated whether the medium used to dilute MAP DNA can affect the activity of Pt compounds, comparing TE buffer and ultrapure water (Figure 2). Ultrapure water enabled a significantly greater reduction of qPCR signal compared to TE buffer for both selected Pt compounds at concentrations of 100 and 1,000 μM. Thus, as expected based on previous findings for other Pt compounds (Busch and Bailar, 1956; Appleton et al., 1982), TE buffer consisting of chelating agent EDTA can chelate Pt compounds used in our study, which subsequently do not bind to DNA and interfere with qPCR. These data demonstrated that EDTA-containing TE buffer should not therefore be used in viability assays utilizing Pt compounds screened herein. As a more suitable dilution medium, we evaluated water which was also used in the study by Soejima et al. (2016), in which DNA as well as a bacterial suspension was diluted with water prior to Pt treatment.

The treatment with two Pt compounds that was shown to suppress amplification of the target sequence of MAP DNA was first optimized for fast-growing *M. smegmatis* as a model for mycobacterial species due to its shorter cultivation time, and finally applied to slow-growing MAP. Based on optimization experiments investigating two Pt compounds at various concentrations, incubation times and temperatures, surfactant utilization and repeated exposures (Figures 3, 4), a procedure for Pt treatment which exhibited maximal suppression of DNA amplification in dead cells and at the same time a minimal impact on the signal from live cells was compiled. This procedure consisted of a single treatment with 100 μM *cis*-dichlorodiammine platinum(II) for 60 min at 5°C. An incubation temperature of 5°C was considered to be more suitable for Pt treatment of mycobacteria than 37°C, which corresponds with the findings of Fraisse et al. (2018), in which the effect of incubation temperature on the treatment of noroviruses with platinum(IV)chloride has also been demonstrated.

The maximum differences achieved between Pt-treated live and dead *M. smegmatis* as well as MAP cells were slightly above 6 Cq (Figures 4, 5), indicating that the Pt treatment did not completely eliminate amplification of the target sequence in dead cells. As in our study, incomplete signal reduction in heat-killed MAP cells (a similar difference of about 7 Cq compared to Pt-treated viable cells) has been reported by Kralik et al. (2010) in a viability assay using PMA dye. In contrast, complete suppression of DNA amplification was achieved using *cis*-dichlorodiammine platinum(II) by previous investigations in enterobacteria by Soejima et al. (2016). The limited effect of both Pt and PMA treatments on mycobacteria could be due to their thicker hydrophobic cell wall rich in mycolic acids responsible for lower penetration of the agents even into dead membrane-compromised cells. Another explanation for the incomplete suppression of DNA amplification in dead mycobacterial cells could be that short DNA sequences (<150 bp) were targeted in qPCR, as compared to the 424 bp amplicon used by Soejima et al. (2016). It has already been reported that the signal suppression from dead cells in viability PCR depends on the amplicon length, i.e., the longer the amplicon size, the more efficient the elimination of the PCR signal (Fittipaldi et al., 2012). On the other hand, a slight qPCR signal reduction after Pt treatment was also observed for non-heat-treated mycobacteria. This can be explained as in the publication by Kralik et al. (2010), in which PMA was used, by the presence of membrane-compromised cells even in an otherwise viable population or by the permeation of Pt compounds into live cells to a limited extent. In addition, Pt molecules are not inactivated after the treatment as occurs with PMA after irradiation and reaction with water molecules (Nocker and Camper, 2009). For this reason, the slight signal reduction for live cells could also be due to the fact that the washing step did not remove all of the Pt molecules contained in the supernatant and these may modify the DNA during the extraction procedure. Our efforts to quantify the initial bacterial suspension of *M. smegmatis* by cultivation were related to this, although the determined cell number varied by about 1.5–2 log_10_ units compared to qPCR, which corresponds to the findings of Kralik et al. (2012). The reduced CFU counts are attributed to the tendency of mycobacterial cells to clump, i.e., one CFU can arise from a cluster of several cells (Pickup et al., 2005). Therefore, the initial cell numbers of *M. smegmatis* as well as MAP were estimated only retrospectively by qPCR.

Since complete suppression of the qPCR signal after Pt treatment for dead cells was not achieved, we investigated whether the use of the surfactant Tween-20 known to affect biological membranes (Burden, 2012) would lead to an increase in signal reduction. Although 0.05% Tween-20 increased the qPCR signal reduction for dead *M. smegmatis* cells, viable cells were also significantly influenced which was an undesirable effect (data not shown). Another study also evaluated the use of Tween-20 (0.5%) in a viral infectivity assay to increase the efficiency of platinum(IV)chloride treatment for noroviruses, but showed no effect (Fraisse et al., 2018).

The mentioned PMA dye was also used in our final experiment, in which its efficiency for distinguishing between viable and dead MAP cells was compared with that of *cis*-dichlorodiammine platinum(II) (Figure 5). According to this experiment, *cis*-dichlorodiammine platinum(II) allowed the discrimination of the MAP cells more efficiently than PMA, which penetrated more markedly into the viable cells. The difference between PMA-treated and untreated viable MAP cells was around 4 Cq, which was remarkably higher compared to the results achieved by Kralik et al. (2010) using the same PMA treatment. The dissimilarity between the results we obtained and those in the above study can probably be explained by the fact that the MAP strain used was more sensitive to PMA treatment. Pt compounds were also evaluated as superior to PMA for discrimination between live and dead bacteria, specifically enterobacteria, by Soejima et al. (2016). Lower cost compared to PMA and no need for activation by light are considered the other advantages of Pt compounds (Soejima et al., 2016).

The last experiment also investigated the effect of the resuspension medium used after Pt and PMA treatment prior to exposing the MAP cells to thermal lysis. Since a DNA amplification decrease was noted for the control sample of dead MAP cells when using ultrapure water as a resuspension medium, we tested TE buffer supplemented with carrier DNA solution (Figure 5), which is known to stabilize DNA and reduce losses during nucleic acid manipulation (Slana et al., 2008), as an additional medium. The use of TE buffer with carrier DNA solution resulted in no difference in Cq values between control samples of live and dead cells, although it did result in a significantly lower difference in Cq values between live and dead MAP cells treated with Pt compound compared to ultrapure water. TE buffer is not, therefore, suitable for use in a viability assay utilizing Pt compounds either as a resuspension medium or as a dilution medium for diluting the cell suspension, as was found in the initial experiment. Heat-killed MAP cells could not rupture during the heat treatment, as this would have the same effect on the cells resuspended with TE buffer with carrier DNA solution, because in both cases the same initial steps were followed, including the same dilution medium. A possible explanation could be that the ultrapure water used does not have the above-mentioned ability to stabilize DNA and reduce DNA losses during manipulation as TE buffer which suppresses the undesired activity of DNases and maintains a stable pH of the solution. Surprisingly, the same effect on the control samples of dead cells as for MAP was not observed for *M. smegmatis*. Further investigation of this matter will, therefore, be needed. However, this outcome does not contradict the desired effect of the Pt compound, i.e., the reduction of the qPCR signal for dead cells with minimal impact on viable cells, as evidenced by the achievement of a comparable ΔCq _dead with Pt – live with Pt_ as in *M. smegmatis*. Similarly, water (sterile) was also used by Soejima et al. (2016) in a viability assay to dilute cell suspensions of enterobacteria and to dissolve the purified DNA. Furthermore, *E. coli* cells were also washed and resuspended in water (distilled) in a viability assay utilizing EMA (Shi et al., 2011). In contrast, ultrapure water was evaluated as an unsuitable dilution medium in the Pt treatment of noroviruses (Fraisse et al., 2018).

## Conclusion

In conclusion, we optimized Pt treatment to assess the viability of mycobacterial cells. The optimal conditions consisted of a single treatment with 100 μM *cis*-dichlorodiammine platinum(II) for 60 min at 5°C. The Pt compound treatment in combination with qPCR demonstrated a better ability to distinguish between viable and dead membrane-compromised mycobacteria suspended in water as compared to PMA. Pt-qPCR therefore proved to be a promising method for the viability determination of MAP cells in foodstuffs and environmental and clinical samples, which could replace time-consuming cultivation or laborious procedures required when using PMA. Further studies could investigate the applicability of the method to other matrices (e.g., milk). As our study addressed only one MAP strain, the following studies could also focus on verifying the optimized procedure in other cattle as well as sheep strains of MAP. It is also necessary to overcome the shortcoming associated with a possible overestimation of viable mycobacterial cell numbers resulting from incomplete elimination of the target sequence amplification in Pt-treated dead cells.

## Data Availability Statement

The original contributions presented in the study are included in the article/supplementary material, further inquiries can be directed to the corresponding author/s.

## Author Contributions

MC designed and performed all the experiments, analyzed and interpreted the data, and wrote the manuscript. MB participated in the initial optimization experiments. VB performed the statistical analysis. IS revised the manuscript. PK designed the study and revised the manuscript. All authors contributed to the article and approved the submitted version.

## Conflict of Interest

The authors declare that the research was conducted in the absence of any commercial or financial relationships that could be construed as a potential conflict of interest.

## Publisher’s Note

All claims expressed in this article are solely those of the authors and do not necessarily represent those of their affiliated organizations, or those of the publisher, the editors and the reviewers. Any product that may be evaluated in this article, or claim that may be made by its manufacturer, is not guaranteed or endorsed by the publisher.
